# Localization of Brain Natriuretic Peptide Immunoreactivity in Rat Spinal Cord

**DOI:** 10.3389/fnana.2016.00116

**Published:** 2016-12-02

**Authors:** Essam M. Abdelalim, Jean-Pierre Bellier, Ikuo Tooyama

**Affiliations:** ^1^Qatar Biomedical Research Institute, Hamad Bin Khalifa University, Qatar FoundationDoha, Qatar; ^2^Molecular Neuroscience Research Center, Shiga University of Medical ScienceOtsu, Japan; ^3^Department of Cytology and Histology, Faculty of Veterinary Medicine, Suez Canal UniversityIsmailia, Egypt

**Keywords:** brain natriuretic peptide, CGRP, ChAT, co-localization, immunohistochemistry, sensory, motor neurons

## Abstract

Brain natriuretic peptide (BNP) exerts its functions through NP receptors. Recently, BNP has been shown to be involved in a wide range of functions. Previous studies reported BNP expression in the sensory afferent fibers in the dorsal horn (DH) of the spinal cord. However, BNP expression and function in the neurons of the central nervous system are still controversial. Therefore, in this study, we investigated BNP expression in the rat spinal cord in detail using reverse transcription-polymerase chain reaction (RT-PCR) and immunohistochemistry. RT-PCR analysis showed that BNP mRNA was present in the spinal cord and dorsal root ganglion (DRG). BNP immunoreactivity was observed in different structures of the spinal cord, including the neuronal cell bodies and neuronal processes. BNP immunoreactivity was observed in the DH of the spinal cord and in the neurons of the intermediate column (IC) and ventral horn (VH). Double-immunolabeling showed a high level of BNP expression in the afferent fibers (laminae I–II) labeled with calcitonin gene-related peptide (CGRP), suggesting BNP involvement in sensory function. In addition, BNP was co-localized with CGRP and choline acetyltransferase (ChAT) in the motor neurons of the VH. Together, these results indicate that BNP is expressed in sensory and motor systems of the spinal cord, suggesting its involvement in several biological actions on sensory and motor neurons via its binding to NP receptor-A (NPR-A) and/or NP receptor-B (NPR-B) at the spinal cord level.

## Introduction

The natriuretic peptide (NP) family consists of atrial NP (ANP), brain NP (BNP) and C-type NP (CNP; Potter et al., 2009). Although BNP was originally discovered in the porcine brain, it is predominately produced from by the heart ventricles (Minamino et al., 1988; Abdelalim et al., 2006a,b). The physiological functions of BNP are induced by its binding to NP receptor-A (NPR-A; Misono et al., 2011). Some studies also suggested that BNP could perform certain functions though it’s binding to NP receptor type B (NPR-B; Suga et al., 1992; Abdelalim and Tooyama, 2009). In response to BNP binding, both guanylyl cyclase receptors produce intracellular cyclic guanosine monophosphate (cGMP; Garbers, 1992). Several studies showed that BNP plays an essential role in cardiovascular homeostasis (Woodard and Rosado, 2007; Potter et al., 2009). However, other reports demonstrated that BNP and its receptors are expressed in several cell types that are not related to cardiovascular control, indicating BNP involvement in several functions (Cameron et al., 1996; Suda et al., 1998; Abdelalim et al., 2007, 2008a,b, 2013; Cao and Yang, 2008; Abdelalim and Tooyama, 2009, 2011a,b).

The expression of NPs was previously detected in the brain and spinal cord of several animals (Zamir et al., 1986; Morii et al., 1987; Ueda et al., 1988; Totsune et al., 1994; Cameron et al., 1996). ANP in the brain is expressed in sensory fibers innervating laminae I–II (Saper et al., 1989). In the spinal cord, ANP and BNP proteins have been found in the fibers of laminae I–II (Kawata et al., 1989; Nohr et al., 1989; Saper et al., 1989). Interestingly, previous reports showed that there are no NP-immunoreactive cell bodies in the spinal cord, suggesting that the immunoreactive fibers might originate from the hypothalamus (Cechetto and Saper, 1988; Nohr et al., 1989). However, our previous study on monkey brain did not detect BNP mRNA in the neurons of the hypothalamus (Abdelalim et al., 2006a). An overview of these findings indicates that, although some studies demonstrated the expression of NPs in the central nervous system, detailed information on the distribution of BNP immunoreactivity in different structures of the spinal cord is lacking. Therefore, in this study, we investigated the distribution of BNP immunoreactivity in different regions of the rat spinal cord. Furthermore, we investigated BNP co-localization with calcitonin gene-related peptide (CGRP) and choline acetyltransferase (ChAT) proteins in sensory and motor systems of the spinal cord.

## Materials and Methods

### Animals and Tissue Preparation

All experimental procedures were approved by the Institutional Animal Care and Use Committee of Shiga University of Medicine, and were designed to minimize the number of animals and their suffering in accordance with the 1996 NIH Guide for the Care and Use of Laboratory Animals.

Nine adult male Wistar rats (Clea Japan, Tokyo, Japan) weighing 200–300 g were used. They were deeply anesthetized by an intraperitoneal injection of sodium pentobarbital (150 mg/kg), and transcardially perfused with 10 mM phosphate-buffered saline (PBS, pH 7.4) at 20°C. For reverse transcription-polymerase chain reaction (RT-PCR) and Western blot analyses, spinal cord segments and dorsal root ganglion (DRG) samples were removed and stored at −80°C. For immunohistochemical studies, rats were similarly anesthetized, perfused transcardially with PBS, and further perfused with an ice-cold fixative of 4% paraformaldehyde in 0.1 M phosphate buffer (pH 7.4). Spinal cord segments were collected and post-fixed by immersion for 24 h in the same fixative at 4°C. After cryoprotection for 4 days in phosphate buffer containing 15% sucrose at 4°C, 20 μm sections were cut in a cryostat. These sections were collected and stored in 0.1 M PBS containing 0.3% Triton X-100 (PBST) at 4°C.

### Reverse Transcription-Polymerase Chain Reaction (RT-PCR)

Total RNA was extracted from DRG and spinal cord samples using a Fastpure RNA kit (Takara Bio, Otsu, Japan) and treated with DNAse (Turbo DNAse, Ambion, Austin, TX, USA) prior to reverse transcription. One microgram of total RNA was reverse-transcribed into cDNA using SuperScript III (Thermo Fisher Scientific Inc., Waltham, MA, USA) and an oligo dT adapter primer. Specific BNP cDNA was amplified by PCR using Ex Taq HS DNA polymerase (Takara Bio) and the following rat BNP primers: 5′-CAGAAGGTGCTGCCCCAGATG-3′ (sense) and 5′-GACTGCGCCGATCCGGTC-3′ (antisense). The amplification profile consisted of an initial step of denaturation at 95°C for 1 min, 36 cycles of 10 s at 98°C, 30 s at 55°C and 1 min at 72°C, followed by final extension at 72°C for 8 min. The PCR products were electrophoresed on a 2% agarose gel and stained with ethidium bromide.

### Western Blot Analysis

Spinal cord samples were homogenized in ice-cold RIPA buffer containing protease inhibitors. The homogenates were centrifuged at 15,000 rpm for 20 min at 4°C, and then the supernatants were collected. Protein concentrations were detected using the Bio-Rad Protein Assay kit (Bio-Rad, Hercules, CA, USA). Protein samples (29.4 μg) were electrophoresed on 15% ready-to-use PAGE (WAKO, Japan) and transferred onto PVDF membranes. The membranes were incubated in 20% reagent N102 (NOF Corp., Tokyo, Japan) in TBS for 1 h at room temperature to block non-specific protein binding sites, then incubated overnight at 4°C with a rabbit polyclonal antibody BNP (Millipore, AB1549), at a dilution of 1:2000 in TBS containing 0.1% Tween-20 (TBST) or with normal rabbit serum (1:2000) in TBST as control. The blots were washed three times for 20 min each with TBST, and then incubated for 1 h at room temperature with peroxidase-conjugated goat anti-rabbit IgG (1:25,000; Jackson ImmunoResearch, Baltimore, PA, USA). After three washes with TBST, the blots were developed using Chemi-lumi One Super (Nacalai, Japan), and visualized using a LuminoImager LAS3000 (FujiFilm, Tokyo, Japan).

### Immunohistochemistry

Serial cryostat sections (20 μm) were floated in 0.1 M PBST (pH 7.4) for 4 days at 4°C and treated in a free-floating state. Endogenous peroxidase activity was blocked by incubating the sections in 0.5% H_2_O_2_ in PBST for 30 min. After washing the sections three times (10 min each) with PBST, they were blocked in 5% bovine serum albumin (BSA) for 2 h at room temperature. After blocking, they were incubated at 4°C for 3 days with a rabbit polyclonal antibody against rat BNP (1:4000 dilution; Millipore, Temecula, CA, USA). The primary antibody was diluted in 2% BSA in PBST. After the sections had been rinsed three times with PBST (10 min each time), they were incubated for 1 h at room temperature with biotinylated goat anti-rabbit IgG (1:3000 dilution; Vector Laboratories, Burlingame, CA, USA) before being rinsed another three times with PBST. The sections were finally incubated for 1 h at room temperature with an avidin-biotin-peroxidase complex (1:4000 dilution; ABC Elite; Vector Laboratories, Burlingame, CA, USA) and the peroxidase-labeled sections were developed in 0.02% 3,3-diamine-benzidine tetrahydrochloride with 0.07% nickel ammonium sulfate in 50 mM Tris-HCl (pH 7.6), with 0.005% H_2_O_2_. Negative control sections were subjected to the same procedures without primary antibody.

### Double-Label Immunofluorescence

Free-floating sections of the spinal cord were blocked for 1 h at room temperature with 5% BSA in PBST to block non-specific binding, then incubated for 48 h at 4°C with a mixture of rabbit polyclonal antibody against rat BNP (diluted 1:2000; Millipore) and either guinea-pig polyclonal CGRP antibody (1:200; T-5053, Peninsula Lab, San Carlos, CA, USA) or goat anti-ChAT antibody (diluted 1:500; Millipore). After three rinses with PBST, sections were incubated for 3 h at room temperature with the appropriate secondary antibodies. The secondary antibodies were Alexa Fluor 555-conjugated anti-rabbit IgG, Alexa Fluor 488-conjugated anti-guinea pig IgG, or Alexa Fluor 488-conjugated anti-goat IgG (diluted 1:500, Thermo Fisher Scientific Inc., Waltham, MA, USA). Negative control sections were subjected to the same procedures without primary antibody. After three washes with PBST, the sections were mounted on gelatin-coated glass slides and examined using a confocal laser-scanning microscope (LSM 510 META, Carl Zeiss, Heidelberg, Germany).

### Image Acquisition and Data Analysis

The major regions of the spinal cord were identified according to the rat brain atlas (Paxinos and Watson, 2006). Images of selected sections were captured and organized into photographic panels. To improve the visualization of the results, only the contrast and brightness of the images were adjusted using Adobe Photoshop, without any further manipulation of the images.

## Results

### Specificity of the BNP Antibody

**Figure 1:**
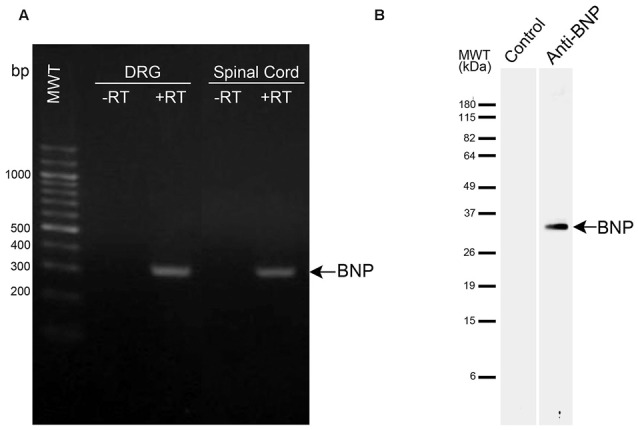
**mRNA and protein expression levels of brain natriuretic peptide (BNP) in the spinal cord. (A)** RT-PCR analysis of BNP expression in DRG and spinal cord. −RT, samples without reverse transcription. BNP band appeared at the expected size (321 bp). **(B)** Western blot analysis was performed using a polyclonal antibody raised against BNP. The antibody recognized a single band at approximately 33 kDa in the spinal cord.

In this study, we used a primary antibody against rat BNP to examine BNP immunoreactivity in the spinal cord. This antibody was previously characterized (Abdelalim and Tooyama, 2009). In our previous study, we confirmed the specificity of this antibody using Western blotting, where it recognized a single band for BNP in rodent cells (Abdelalim and Tooyama, 2009). Furthermore, in the current study we performed Western blotting to confirm the specificity on rat spinal cord samples. A specific band was detected at approximately 33 kDa in the spinal cord samples, corresponding to BNP protein (Figure 1B). It is known that Western blotting is effective for validating antibody specificity (Burry, 2000) and thus the antibody used in this study can be considered to recognize rat BNP protein specifically.

### Expression of BNP mRNA in the Rat Spinal Cord and DRG

RT-PCR was used to examine the expression of BNP mRNA in the spinal cord and DRG of rat. This analysis showed a single band for BNP at the expected size (321 bp) in the DRG and spinal cord (Figure 1). Here we included the DRG in the examination because DRG neurons are known to send projections to the dorsal horn (DH) of the spinal cord, which showed BNP immunoreactivity in the current study.

### Distribution of BNP Immunoreactivity in the Rat Spinal Cord

**Figure 2:**
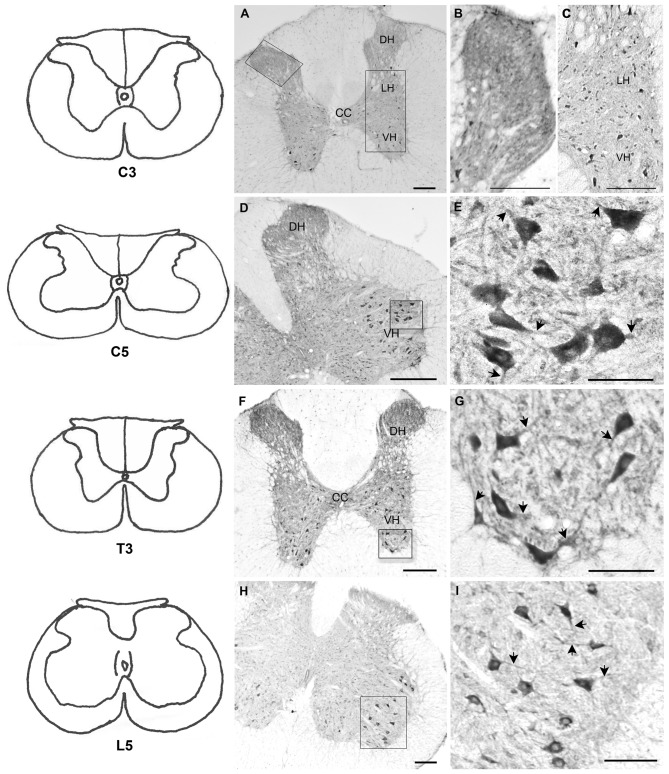
**Localization of BNP protein in rat spinal cord.** Left panels, line drawings for all segments presented in the figure (C3, C5, T3 and L5) based on the rat brain atlas (Paxinos and Watson, 2006).** (A)** BNP immunoreactivity in C3 spinal segment. Note that BNP immunoreactivity was distributed throughout the gray matter. **(B)** High intensity of BNP-immunoreactive fibers in the dorsal horn (DH) of C3. **(C)** BNP-immunoreactive neurons in the ventral horn (VH). **(D)** BNP immunoreactivity in a caudal segment of the cervical spinal cord (C5). The immunoreactivity was seen in the DH, intermediate column (IC) and VH. **(E)** A high-magnification image shows BNP immunoreactivity in the perikarya and neuronal processes (arrowheads) in the VH. **(F)** BNP immunoreactivity in a thoracic segment of the spinal cord (T3). The immunoreactivity was seen in the DH, IC and VH. **(G)** A high-magnification image shows BNP immunoreactivity in the perikarya and neuronal processes (arrowheads) in the VH. **(H)** BNP immunoreactivity in a lumber segment of the spinal cord (L5). The immunoreactivity was seen in the DH, IC and VH. **(I)** A high-magnification image shows BNP immunoreactivity in the perikarya and neuronal processes (arrowheads) in the VH. Scale bars in **(A–I)** = 100 μm.

Immunohistochemical analysis revealed BNP immunoreactivity in different regions of the spinal cord (cervical, thoracic, lumbar and sacral) in neuronal cell bodies and nerve fibers (Figure 2). In the negative control sections, no immunoreactivity was observed (data not shown). In the cervical spinal cord segments, BNP-immunoreactive dots were detected throughout the laminae in the gray matter (Figures 2A–C). In the DH, high-intensity of BNP-immunoreactive fibers was noted in the DH (Figures 2A,B). In addition, the immunoreactive dots were evident near the spinal canal (Figures 2A,C). The BNP-immunoreactive cell bodies were detected in the cervical segments and continued throughout the spinal cord (Figures 2A–I). In the DH, BNP immunoreactivity was also seen in nerve fibers (Figures 2D,F). BNP-immunoreactive neurons were clearly observed in the intermediate column (IC) and the ventral horn (VH) of the spinal cord (Figures 2A,C–I). In the VH, strong BNP immunoreactivity was seen in the perikarya and dendrites of the multipolar neurons, indicating its localization in the motor spinal neurons (Figures 2C,E,G,I).

### Double-Label Immunostaining

**Figure 3:**
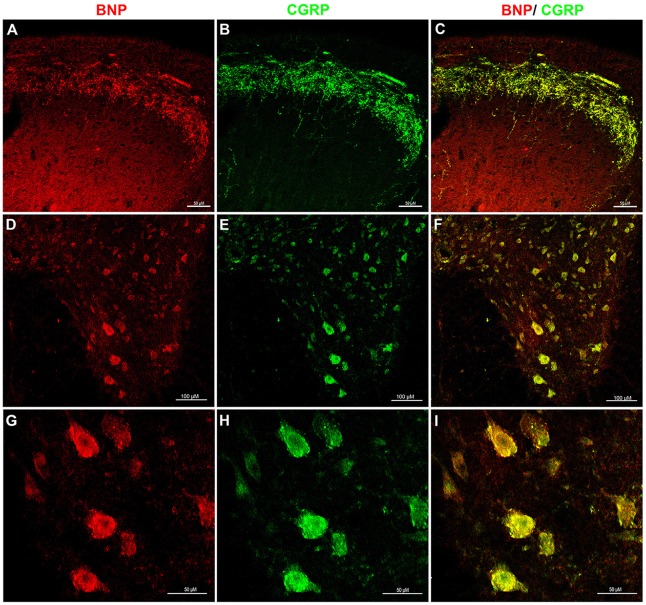
**Immunofluorescence confocal photomicrographs, showing the co-localization of BNP and calcitonin gene-related peptide (CGRP) in rat spinal cord.** Spinal cord sections were labeled for BNP (red) and CGRP (green). **(A)** BNP immunoreactivity in the DH of the spinal cord. **(B)** CGRP-immunoreactive fibers in laminae I–II. **(C)** Merged images show the co-localization of BNP and CGRP in superficial laminae I–II. **(D)** A low-magnification image shows BNP immunoreactivity in the VH. **(E)** A low-magnification image shows CGRP immunoreactivity in the VH. **(F)** Merged images the co-localization of BNP and CGRP in the VH. **(G)** A high-magnification image shows BNP-immunoreactive neurons in the VH. **(H)** A high-magnification image shows CGRP-immunoreactive motor neurons in the VH. **(I)** Merged images show the co-localization of BNP and CGRP in the VH motor neurons. Scale bars in **(A–C)** and **(G–I)** = 100 μm and in **(D–F)** = 50 μm.

To investigate whether BNP is co-localized with CGRP in sensory and motor systems in the spinal cord, we performed a confocal microscopic analysis of the rat spinal cord sections to recognize CGRP and BNP co-localization. It has been shown that CGRP is expressed in the afferent fibers of laminae I–II of the DH (Eftekhari and Edvinsson, 2011). Double-immunolabeling of BNP and CGRP showed that BNP immunoreactivity was co-localized with CGRP in the DH of the spinal cord, indicating BNP expression in laminae I–II (Figures 3A–C). Furthermore, in the VH, BNP was co-localized with CGRP in the perikarya of the motor neurons (Figures 3D–I).

**Figure 4:**
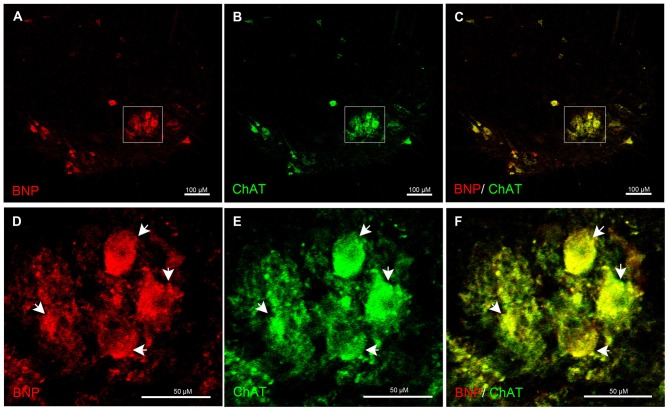
**Immunofluorescence confocal photomicrographs, showing the localization of BNP (red) in motor neurons (green) of the rat spinal cord.** BNP-labeled cells in the VH **(A)**. **(B)** Choline acetyltransferase (ChAT)-labeled cells in the VH. **(C)** Merged image shows the co-localization of BNP and ChAT in the motor neurons. **(D–F)** A higher-magnification image shows the co-localization of BNP and ChAT in the motor neurons (arrowheads). Scale bars in **(A–C)** = 100 μm and in **(D–F)** = 50 μm.

Furthermore, to examine whether spinal motor (ChAT-positive) neurons express BNP, we performed a laser confocal microscopic analysis of the rat spinal cord to recognize BNP and ChAT co-localization. It is known that ChAT is a marker for motor neurons, which are present in the VH of the spinal cord. BNP immunoreactivity was co-localized with ChAT-positive neurons in the VH of the spinal cord, indicating BNP expression in the rat spinal motor neurons (Figures 4A–F).

## Discussion

In the current study, we have revealed the detailed distribution of BNP immunoreactivity in the rat spinal cord. We have also reported for the first time that BNP protein is co-localized with CGRP and ChAT proteins in the sensory and motor systems of the spinal cord.

Previous studies illustrated the localization of BNP in the spinal cord in the fibers of laminae I–II (Kawata et al., 1989; Saper et al., 1989; Zhang et al., 2010). Earlier reports showed that there are no NP-immunoreactive perikarya in the spinal cord and suggested that the immunoreactive fibers may originate from the hypothalamus (Cechetto and Saper, 1988; Nohr et al., 1989). However, our recent study did not detect BNP immunoreactivity in the neurons of the monkey hypothalamus (Abdelalim et al., 2006a). BNP expression in the spinal cord neurons and its function remain controversial. Thus, to provide evidence for a putative role of BNP in the spinal cord, we used a specific antibody to investigate the distribution of BNP immunoreactivity in different regions of the rat spinal cord and its co-localization with ChAT and CGRP proteins. In the spinal cord, we detected BNP immunoreactivity in the neuronal cell bodies in the IC and VH throughout the spinal cord, suggesting its expression in the interneurons and motor neurons.

### Localization of BNP in Sensory Afferent Fibers of the Dorsal Horn

In the current study, we detected BNP immunoreactivity in the DH of the spinal cord. Furthermore, a double-immunostaining experiment showed that BNP protein is co-localized with CGRP protein in the DH of the spinal cord. CGRP is released from primary afferent fibers that convey nociceptive information in the spinal cord and plays a crucial role in pain sensitivity (Gangadharan and Kuner, 2013). The expression profile of BNP with CGRP in the DH of the spinal cord suggests that BNP is expressed in the primary afferent terminals of nociceptive DRG neurons. This was confirmed in this study using RT-PCR, where we found that BNP mRNA is also expressed in rat DRG.

Our results agree with earlier studies, in which BNP immunoreactivity was detected in laminae I–II of rat and pig (Kawata et al., 1989; Saper et al., 1989). However, a recent study was able to detect BNP protein in the spinal DH using Western blotting, but could not detect BNP immunoreactivity in the afferent fibers using immunohistochemistry (Zhang et al., 2010). These variations are due to technical differences. In our study, we used very sensitive protocols to detect BNP immunoreactivity. As described above, the rat tissues were fixed using a perfusion system and the immunohistochemical procedure was performed using a free-floating technique, which is more sensitive than other immunohistochemical techniques.

BNP and its receptors (NPR-A and NPR-B) are expressed in rat DRG (Zhang et al., 2010; Abdelalim et al., 2013) and it has been found that their expressions are increased in response to pain induction (Zhang et al., 2010). The BNP protein has been found in CGRP-and isolectin B4 (IB4)-positive DRG neurons (Zhang et al., 2010). Interestingly, our recent study showed the co-localization of NPR-B with CGRP in the same DRG neurons and in the DH of the spinal cord (Abdelalim et al., 2013). It is known that CGRP is expressed in DRG small neurons and afferent fibers in laminae I–II of the spinal cord (Gibson et al., 1984; Schaible, 1996). After acute pain induction in animal models, CGRP level is elevated in the DRG and DH of the spinal cord (Traub et al., 1990; Zhang et al., 1993). Transection of the DH causes a dramatic decrease of the CGRP expression in the laminae I–II in the DH (Fernandez et al., 2003). However, brachial plexus injury leads to an increase in the expression of CGRP in the DH (Chen et al., 2010). These findings indicate the essential role of CGRP in the pain pathway and its level is affected by the nature and type of the nerve injury (Bergman et al., 1996; Li et al., 2004). Taken together, these findings suggest that BNP in the DRG and the spinal cord may be involved in pain modulation through NPR-A and/or NPR-B receptors.

### Localization of BNP in Spinal Cord Motor Neurons

The current study is the first to examine the relationship between BNP and motor neurons in the spinal cord. We found that BNP was co-localized with CGRP in the multipolar neurons in the VH. Our results are consistent with those of the previous studies, which showed that the CGRP is expressed in the motor neuron in the ventral of the spinal cord (Chen et al., 2010; McCoy et al., 2012; Cui et al., 2015; Kim et al., 2016), where it is co-localized with ChAT (Zheng et al., 2008). CGRP plays different roles based on its localization. It has been reported that the expression level of CGRP in the motor neurons changes after nerve injury and these changes depend on the age of the animal and the type of the injury. In the spinal cord, CGRP immunoreactivity is temporarily elevated in laminae I–IV of the DH and motor neurons of the VH after suffering a sciatic nerve injury (Zheng et al., 2008). Another study reported that sciatic nerve injury leads to down-regulation of CGRP transcripts in the motor neurons of the VH in the first week after birth and starts to be unregulated 12 days after birth (Tonra and Mendell, 1998). Recently, it has also been suggested that CGRP expression in the spinal cord is involved in the growth of sensory and motor axons (Kim et al., 2016). Taken together, these findings suggest that BNP might play a role in repair mechanisms following nerve injuries.

We also found that BNP was co-localized with ChAT-positive neurons, indicating its localization in the spinal motor neurons in the VH. Our previous report demonstrated the co-localization of NPR receptors (NPR-A, NPR-B and NPR-C) with ChAT-positive neurons in the central nervous system (Abdelalim et al., 2007, 2008a,b; Abdelalim and Tooyama, 2010). The relationship between NPs and cholinergic (ChAT-positive) neurons has been previously reported. It has been found that stimulation of cholinergic neurons in the anteroventral third ventricle leads to induction of ANP expression hypothalamus (Antunes-Rodrigues et al., 1996). These findings suggest that NPs may play an essential role in mediating motor function by binding to their receptors. These findings also indicate that BNP may be involved in specific motor functions in the spinal cord.

## Conclusion

In the current study, we detected BNP immunoreactivity in the afferent fibers in the DH of the spinal cord, as previously reported, and showed that this immunoreactivity was co-localized with CGRP protein, suggesting BNP involvement in pain mechanisms. Interestingly, we detected BNP immunoreactivity in the neurons of the IC and VH of the spinal cord. Using double immunostaining, we showed that BNP is co-localized with CGRP and ChAT in the VH of the spinal cord, indicating its expression in motor neurons. The main findings of this report are as follows: (1) This is the first report to confirm the localization of BNP in the spinal neurons, particularly motor neurons. (2) This is the first time the relationship between BNP and CGRP or ChAT in the spinal cord has been studied. Taking these findings together with previous studies, BNP expression in the primary sensory afferents and motor neurons suggests its involvement in the pain pathway and motor function through its binding to NPR-A and/or NPR-B receptors. Further functional studies are needed to clarify the exact role of BNP in the sensory and motor systems of the spinal cord.

## Author Contributions

EMA: study concept and design; EMA and J-PB: acquisition of data; EMA, J-PB and IT: analysis and interpretation of data; EMA: wrote the manuscript; EMA, J-PB and IT: revision of manuscript.

## Conflict of Interest Statement

The authors declare that the research was conducted in the absence of any commercial or financial relationships that could be construed as a potential conflict of interest. The reviewer SW and handling Editor declared their shared affiliation, and the handling Editor states that the process nevertheless met the standards of a fair and objective review.
